# Supplementary figures and images for: Immunogenicity Characterization of the Recombinant gI Protein Fragment from Pseudorabies Virus and an Evaluation of Its Diagnostic Use in Pigs
Source: Vet Sci. 2023 Aug 5;10(8):506. doi: 10.3390/vetsci10080506 (PMC10458116; doi:10.3390/vetsci10080506)

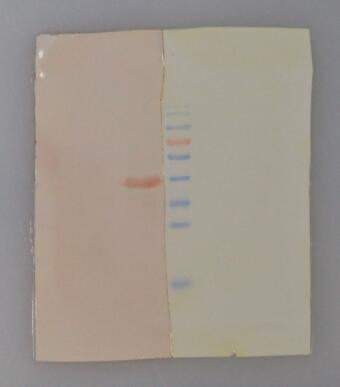

Supplement: Supplementary file 1 [file vetsci-10-00506-s001.zip › vetsci-2526046-supplementary.jpg]
